# Standardising the management of open extremity fractures: a scoping review of national guidelines

**DOI:** 10.1007/s00590-022-03324-w

**Published:** 2022-07-10

**Authors:** Juan Enrique Berner, Stephen R. Ali, Patrick A. Will, Rodrigo Tejos, Jagdeep Nanchahal, Abhilash Jain

**Affiliations:** 1 Kellogg College, University of Oxford, Oxford, UK; 2 Plastic Surgery Department, The Royal London Hospital, Barts Health NHS Trust, Whitechapel Rd., E1 1FR, London, UK; 3 Reconstructive Surgery and Regenerative Medicine Research Group, Institute of Life Sciences, Swansea University Medical School, Swansea, UK; 4 Welsh Centre for Burns and Plastic Surgery, Morriston Hospital, Swansea, UK; 5 BG Klinik Ludwigshafen, Ludwigshafen Am Rhein, Germany; 6 Ruprecht Karl University of Heidelberg, Heidelberg, Germany; 7 Sección de Cirugía Plástica y Reconstructiva. Escuela de Medicina, Pontificia Universidad Católica de Chile, Santiago, Chile; 8 The Kennedy Institute of Rheumatology, Nuffield Department of Orthopaedic, Rheumatology and Musculoskeletal Sciences, University of Oxford, Oxford, UK; 9 Imperial College Healthcare NHS Trust, London, UK; 10 Nuffield Department of Orthopaedic, Rheumatology and Musculoskeletal Sciences, University of Oxford, Oxford, UK

**Keywords:** Open fractures, Guidelines, Lower limb, Trauma, Open fractures, Lower extremity, Plastic surgery

## Abstract

**Introduction:**

Open extremity fractures can be life-changing events. Clinical guidelines on the management of these injuries aim to standardise the care of patients by presenting evidence-based recommendations. We performed a scoping systematic review to identify all national clinical practice guidelines published to date.

**Materials and methods:**

A PRISMA-compliant scoping systematic review was designed to identify all national or federal guidelines for the management of open fractures, with no limitations for language or publication date. EMBASE and MEDLINE database were searched. Article screening and full-text review was performed in a blinded fashion in parallel by two authors.

**Results:**

Following elimination of duplicates, 376 individual publications were identified and reviewed. In total, 12 clinical guidelines were identified, authored by groups in the UK, USA, the Netherlands, Finland, and Malawi. Two of these focused exclusively on antibiotic prophylaxis and one on combat-related injuries, with the remaining nine presented wide-scope recommendations with significant content overlap.

**Discussion:**

Clinical practice guidelines serve clinicians in providing evidence-based and cost-effective care. We only identified one open fractures guideline developed in a low- or middle-income country, from Malawi. Even though the development of these guidelines can be time and resource intensive, the benefits may outweigh the costs by standardising the care offered to patients in different healthcare settings. International collaboration may be an alternative for adapting guidelines to match local resources and healthcare systems for use across national borders.

## Introduction

The aim of developing clinical guidelines is to improve the quality of care for patients by providing clinicians with cost-effective, evidence-based recommendations [1]. The production of these documents usually involves a collaborative effort, including the conduction of multiple systematic reviews, commonly orchestrated by governmental bodies and scientific societies. Advocates for the use of clinical guidelines claim that by making these recommendations widely available, guidelines promote standardisation of practice for clinical conditions that might otherwise be managed in heterogeneous and non-evidence-based ways [2]. Furthermore, guidelines can provide a benchmark for quality control and continuous audit of best practices.

Open extremity fractures are severe injuries, involving traumatic loss of skeletal continuity with associated disruption of the surrounding soft tissues. These can vary in severity depending on the amount of energy involved, level of contamination and tissue damage, with the potential for permanent loss of form and function for patients. For severe Gustilo IIIB and IIIC fractures, a combined amputation rate of 7.3% has been reported [3]. Likewise, the LEAP study concluded that even 2 years post-injury, 10.9% of their cohort developed non-union of the fracture and 3.9% had non-healed wounds [4]. Open fractures also adversely affect the mental health of patients, with reported quality of life equivalent to death in the early stages post-injury [5].

In order to optimise the management of patients presenting after sustaining complex extremity trauma, direct transfer to specialist centres capable of providing multidisciplinary interventions has been advocated [6, 7]. This allows timely involvement of orthopaedic and plastic surgeons, microbiologists, radiologists, and physiotherapists with an interest in major trauma, aiming for early skeletal stabilisation and soft tissue coverage of the fracture site, leading to better outcomes [8].

Clinical guidelines focusing on the management of open fractures aim to streamline and standardise the management of patients with complex extremity trauma by presenting a series of recommended interventions that should take place within defined timescales. Enforcement of guidelines ensures that patients admitted for open fractures receive an evidence-based and cost-effective package of care, reducing disparities within health systems [9].

Several countries have developed their own national guidelines and others have attempted to do so but have been forced to abandon the process due to lack of resources. There have been no previous studies on the number of clinical guidelines for lower limb open fractures available to date, nor their scope, contents, and methodology. Considering that trauma is a global concern with a burden that is even worse in low-income economies [10], we performed a systematic scoping review to identify all national standards or regional standards for managing open extremity injuries. This should provide a foundation for future appraisals [11] and foster of international collaboration.

## Materials and methods

This review followed the principles of the PRISMA statement and its extension for scoping reviews [12, 13]. A study protocol was registered prospectively on the Open Science Framework, including exclusion and inclusion criteria (Table 1), screening and data management pathways. Only national open extremity open fractures guidelines or publications that referred or cited these were deemed eligible for inclusion. Narrative and systematic reviews on the management of open fractures, and personal or institutional recommendations were excluded.

**Table 1:** Inclusion and exclusion criteria for systematic scoping review

Inclusion	Exclusion
National or regional clinical guidelines on the management of open extremity fractures.	Local or single-institution guidelines
Publications referencing national or regional clinical guidelines	Narrative or systematic reviews on the management of open fractures

A senior librarian with experience in systematic reviews aided in the design of a search strategy, using the following search terms: “open fracture”, “compound fracture”, “guideline”, “consensus”, “recommendation” and “standards” (Appendix). Searches were conducted into EMBASE and MEDLINE databases on the 10th of February 2021 without any filters or limitations in terms of language or publication date. Abstract and conference proceedings were also included.

Mendeley Desktop (Elsevier. London, United Kingdom) was utilised for identification and conciliation of duplicate entries. Parallel and blinded screening of titles and abstracts was conducted by two authors (JB and SRA) using Rayyan QCRI software [14] (Qatar Computing Research Institute, Qatar). This was followed by full text and references review to identify eligible studies, as per the pre-stablished inclusion and exclusion criteria. AJ was nominated for addressing and resolving discrepancies among reviewers. Eligible publications were reviewed, again in parallel and independently, by two authors (SRA and PW) using a pre-defined data gathering spreadsheet to allow identification of national guidelines for open fractures. Each guideline was reviewed to identify scope of included recommendations.

## Results

**Fig. 1:**
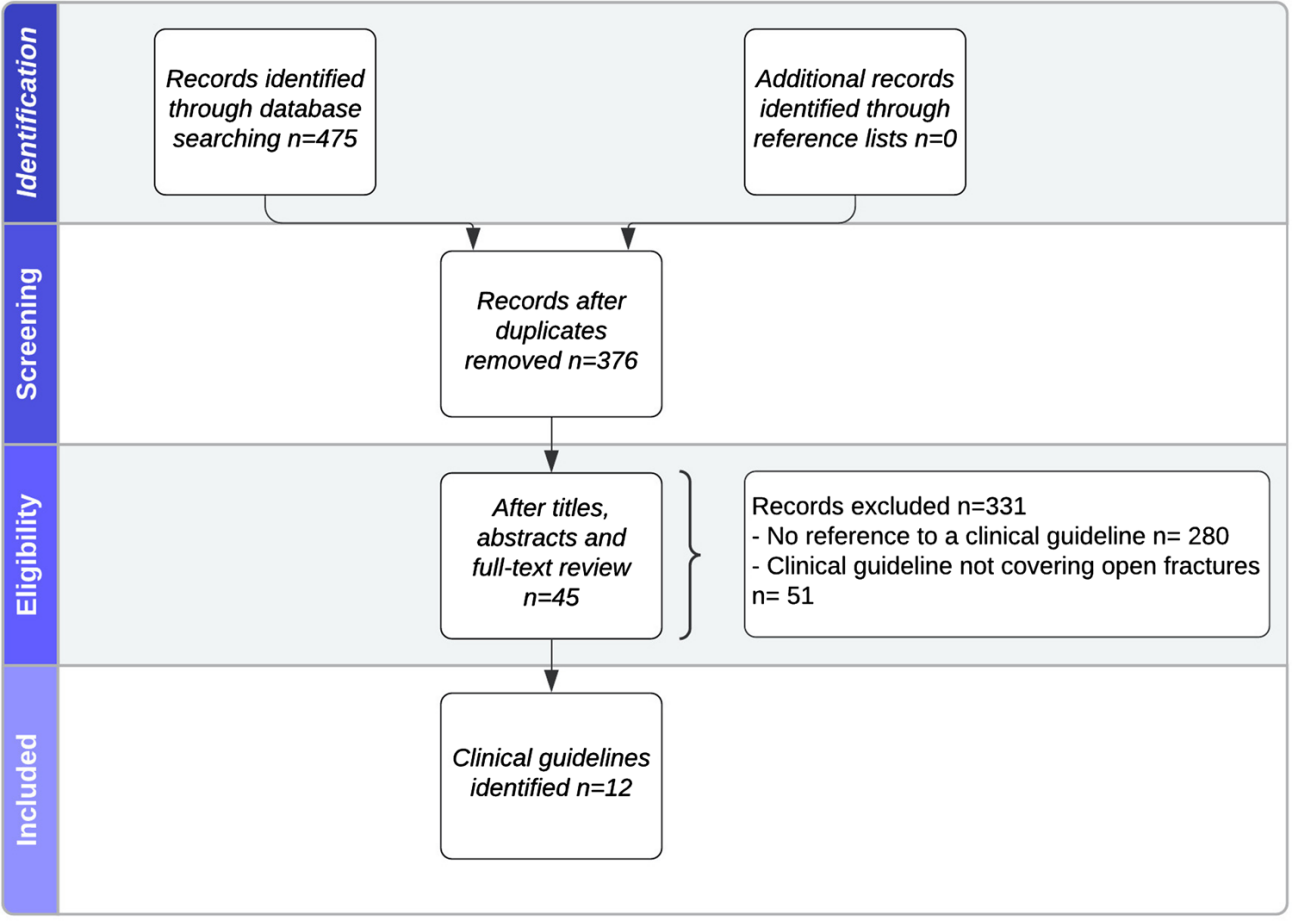
PRISMA flow-diagram

A total of 475 publications were identified. Of these, 215 were retrieved from MEDLINE and 260 from EMBASE. Ninety-nine entries were identified as duplicate, resulting in 376 articles for further review. Following title, abstract and full-text review, 45 entries presented or referred to a national guideline for open fracture management (Fig. 1).

Eligible studies included manuscripts published from 1997 to date, of which 40 were written in English, 3 in German, and 2 in Finnish. Six provided guidance for the management of open fractures, while the remaining 39 publications made reference to at least one. Overall, 12 clinical guidelines for the treatment of open fractures were identified (Table 2).

**Table 2:** National guidelines identified in systematic scoping review

	Title	Responsible organisation	Country	Year of publication
1	A report by the British orthopaedic association/British association of plastic surgeons working party on the management of open tibial fractures [16]	British orthopaedic association (BOA) and British association of Plastic surgeons (BAPS)	United Kingdom	1997
2	[Guidelines for the treatment of tibial fractures of adult patients] [21]	Finnish orthopaedic society	Finland	2004
3	Prophylactic antibiotic use in open fractures: an evidence-based guideline [13]	Surgical infection society	United States of America	2006
4	Standards for the management of Open fractures of the lower limb [17]	British orthopaedic association (BOA) and British association of plastic, reconstructive and aesthetic surgeons (BAPRAS)	United Kingdom	2009
5	Guidelines for the prevention of infections associated with combat-related injuries: 2011 update [15]	Infectious diseases society of America and the surgical infection society	United States of America	2011
6	East practice management guidelines work group: update to practice management guidelines for prophylactic antibiotic use in open fractures [14]	Eastern association for the surgery of trauma (EAST)	United States of America	2011
7	[Update on current care guidelines: treatment of tibial shaft fractures] [22]	Finnish orthopaedic society	Finland	2011
8	Best practices in the management of orthopaedic trauma [20]	American college of surgeons and orthopaedic Trauma association	United States of America	2015
9	Fractures (complex): assessment and management [19]	The national institute for health and care excellence (nice)	United Kingdom	2016
10	Open fractures of the lower limb [23]	Federatie medisch specialisten	The Netherlands	2017
11	Standards for the management of open fractures [24]	British orthopaedic association (BOA) and British association of plastic, reconstructive and aesthetic surgeons (BAPRAS)	United Kingdom	2020
12	The Malawi orthopaedic association/AO alliance guidelines and standards for open fracture management in Malawi [18]	Malawi orthopaedic association and AO Alliance	Malawi	2020

Two of these guidelines covered recommendations for antibiotic prophylaxis only [15, 16], while one focused on the management of combat-related injuries [17], all three being developed by organisations based in the USA. The remaining nine covered the overall management of open fractures. Of these, three were the result of collaboration between the British Association of Plastic, Reconstructive and Aesthetic Surgeons (BAPRAS) and the British Orthopaedic Association (BOA), published in 1997 [18], 2009 [19], and 2020 [20]. A further guideline was developed by the United Kingdom National Institute for Health and Care Excellence (NICE) [21]. The American College of Surgeons Trauma Quality Programs Guideline on the Management of Orthopaedic Trauma was also included [22], along with two iterations of the guidelines developed by the Finnish Orthopaedic Association on the management of tibial fractures published in 2004 [23] and 2011 [24]. The national guidelines for the Netherlands [25] and Malawi [20] published in 2017 and 2020, respectively, were also included.

**Fig. 2:**
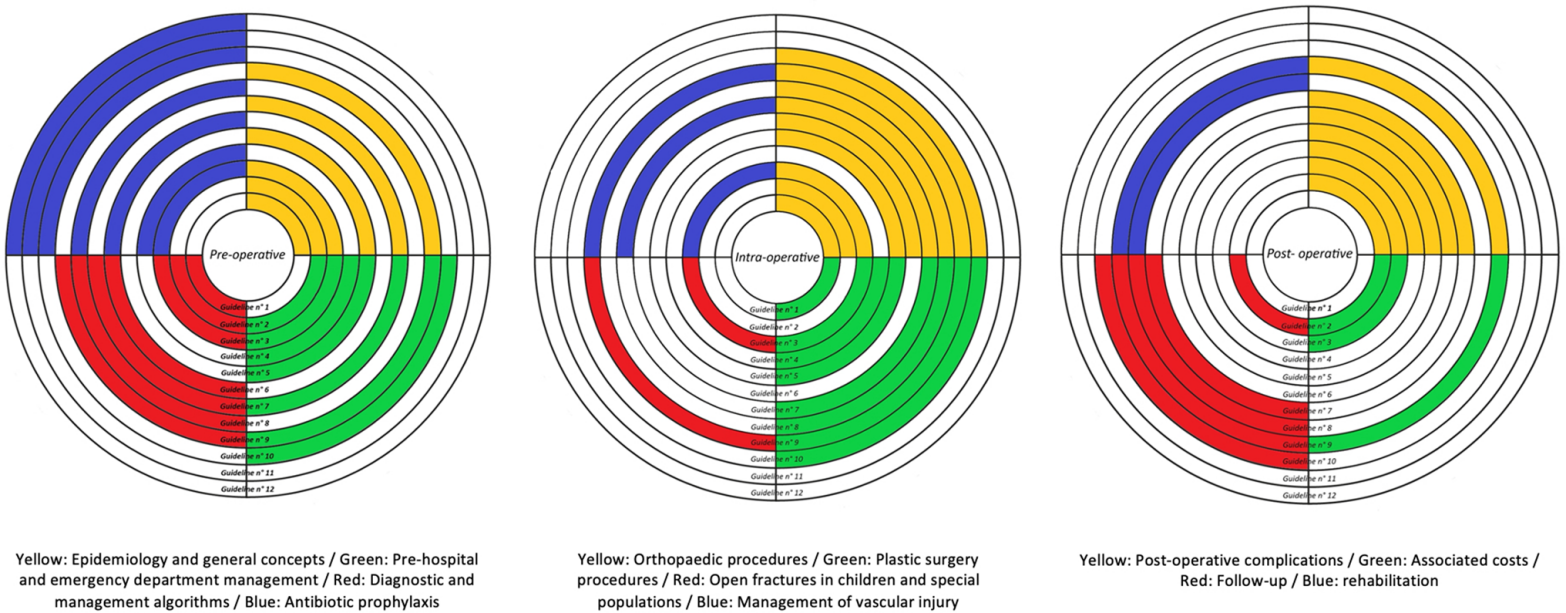
Diagram showing the overlap in contents covered by the guidelines identified in this systematic scoping review

The contents of each guideline are shown in Table 3. We found extensive overlap in the scope of the guidelines in terms of pre-operative, surgical and post-operative management of open extremity fractures (Fig. 2).

**Table 3:** Table of contents for each clinical practice guideline identified in scoping review

	Title	Contents
1	A report by the british orthopaedic association/british association of plastic surgeons working party on the management of open tibial fractures – UK, 1997 [16]	Epidemiology of open fractures
		Injury recognition
		Communication
		Timing
		Pre-operative management
		The first orthopaedic procedure
		Safe incisions and fasciotomy
		Commonly used methods of soft tissue reconstruction
2	[Guidelines for the treatment of tibial fractures of adult patients]—Finland, 2004 [21]	Incidence and methods of treatment
		Definition and soft tissue injuries classification
		Choice of treatment setting
		First aid
		Assessment and diagnosis
		Principles for treating tibial fractures and treatment modality selection
		Intramedullary nailing
		Follow-up treatment
		Plate fixation
		Conservative treatment
		External fixation
		Treatment of severe open fractures
		Centralisation of specialist centres
		Treatment of tibial fractures in high-risk patients
		Complications
		Costs
		Evaluation criteria
3	Prophylactic antibiotic use in open fractures: An evidence-based guideline—USA, 2006 [13]	Prophylactic antibiotics
4	Standards for the management of open fractures of the lower limb—UK, 2009 [17]	Specialist centres for complex open lower limb fractures
		Primary management in the emergency department
		Antibiotic prophylaxis
		Timing of wound excision in open fractures
		Guidelines for wound debridement (excision)
		Bone exposure, decontamination and preservation: debridement
		Degloving
		Classification of open fractures
		Temporary wound dressings
		Techniques for skeletal stabilisation in open tibial fractures
		Timing of soft tissue reconstruction
		Type of soft tissue reconstruction
		Compartment syndrome
		Vascular injuries
		Open fractures of the foot and ankle
		When things go wrong with soft tissues
		When things go wrong with bone
		Guidelines for primary amputation
		Outcome measures
		Management of severe open fractures in children
5	Guidelines for the prevention of infections associated with combat-related injuries: 2011 update—USA, 2011 [15]	Initial care in the field
		Prophylactic antibiotics
		Debridement and irrigation
		Surgical wound management
		Facility infection control and prevention
6	East practice management guidelines work group: update to practice management guidelines for prophylactic antibiotic use in open fractures—USA, 2011 [14]	Antibiotic prophylaxis
7	[Update on current care guidelines: treatment of tibial shaft fractures]—Finland, 2011 [22]	First aid
		Research
		Intramedullary nailing
		Plate fixation
		Plaster treatment
		Treatment of severe fractures
		Complications
		Non-union
8	Best Practices in the Management of Orthopaedic Trauma—USA, 2015 [20]	Prophylactic antibiotics
		Open fractures
		Damage control orthopaedic surgery
		The mangled extremity
		Compartment syndrome
9	Fractures (complex): assessment and management—UK, 2016 [19]	Initial management of open fractures before debridement
		Splinting long bone fractures of the leg in pre-hospital setting
		Destination for people with suspected fractures
		Vascular injury in hospital settings
		Compartment syndrome
		Whole-body CT of multiple injuries
		Management of open fractures before debridement
		Limb salvage in people with open fractures
		Debridement, staging of fixation and cover
		Documentation
10	Open Fractures of the Lower Limb—Netherlands, Best Practices in the Management of Orthopaedic Trauma—Netherlands, 2017 [23]	Diagnosis and treatment of open limb fractures
		Debridement of open limb fractures
		Provisional treatment of soft tissue injury
		Internal fixation of open limb fractures
		Cancellous bone grafting open limb fracture
		Definitive treatment of soft tissue injuries
		Amputation of open limb fracture
		Postoperative antibiotics open limb fractures
		Exercise therapy after open limb fractures
		Organisation of care open limb fractures
		Patient communication
11	British association of plastic, reconstructive and aesthetic surgery (BAPRAS) and british orthopaedic association (BOA) standards for the management of open fractures—UK, 2020 [24]	Prehospital and emergency department care, including prophylactic antibiotics
		Timing of wound excision
		Wound excision
		Degloving injuries
		Temporary wound dressings
		Skeletal stabilisation
		Timing of soft tissue reconstruction
		Soft tissue reconstruction
		Bone loss in open fractures
		Ascular injuries
		Compartment syndrome in the lower limb
		Amputation
		Infection
		Open tibial fractures in children
		Open fragility fractures
		Outcome measures
		Patient experience of open fracture and practical Psychological support
		Rehabilitation after severe open tibial fractures
		Special circumstances: blast, ballistics, and mass casualties
		Setting up an effective orthoplastic service
12	The Malawi orthopaedic association/AO alliance guidelines and standards for open fracture management in Malawi–Malawi, 2020 [18]	ATLS
		Prophylactic antibiotics
		Primary management in emergency department
		Timing of debridement
		Timing of soft tissue reconstruction
		Timing of skeletal stabilisation
		Amputation
		Documentation

## Discussion

Our systematic scoping review identified a total of 12 clinical guidelines for open fractures from only five countries, Finland [23, 24], Malawi [20], the Netherlands [25], UK [18, 19, 21, 26], and the USA [15–17, 22]. With the exception of two guidelines on antibiotic prophylaxis [15, 16], and another focusing on battlefield-related injuries[17], most of the others provided a comprehensive set of recommendations, including pre-hospital management, referral to specialist centre, management in the emergency department, along with timing and modalities for skeletal fixation and soft tissue closure.

The first open fracture national guideline to be published was the 1997 British Association of Plastic Surgeon and British Orthopaedic Association working party report[18]. The British Societies have published more comprehensive guidelines on two subsequent occasions, in 2009[19] and 2020 [26]. While the Finnish Orthopaedic Association and American College of Surgeons standards had their own independent inception, the Dutch and Malawian guideline working parties were influenced by the British experience and, therefore, follow a similar scope and structure.

To our knowledge, this is the first systematic review to investigate published clinical guidelines for open fractures. By following the PRISMA guidelines [12, 13] and conducting a broad systematic search with no limits or filters, we intended to capture all the guidelines published to date. However, since we could only identify indexed guidelines and guidelines being referenced by an indexed article, it is possible that there are other guidelines.

Clinical practice guidelines provide clinicians with recommendations based on the best available evidence at the time of writing [1]. These allow standardisation of the quality of care provided, regardless of geographical location and financial situation, reducing the risk for inequality in health systems. Even though poor-quality guidelines have been identified, potentially misleading clinicians towards non-cost-effective interventions [27], specialised institutions such as the National Institute for Health and Care Excellence in the UK and the Federation of Medical Specialties in the Netherlands have professionalised the development of clinical guidelines to assure their quality.

Our search strategy only identified a clinical guideline for open fracture from one developing country, Malawi. The development of robust evidence-based national guidelines is resource and time consuming. Given the considerable overlap in scope and content, we propose that adaptation of existing guidelines considering local resources and healthcare systems would not only be far more cost effective but would also foster international collaboration. It would be challenging to develop a single international guideline given the wide disparity between different countries.

We did not assess quality of the development of each guideline or their recommendations as they spanned 25 years and evolved with the development of evidence and methodology. Future studies comparing contemporaneous guidelines in different territories would highlight differences in development methodologies and how recommendations could be adapted across national borders.

## Figures and Tables

**Fig. 3:**
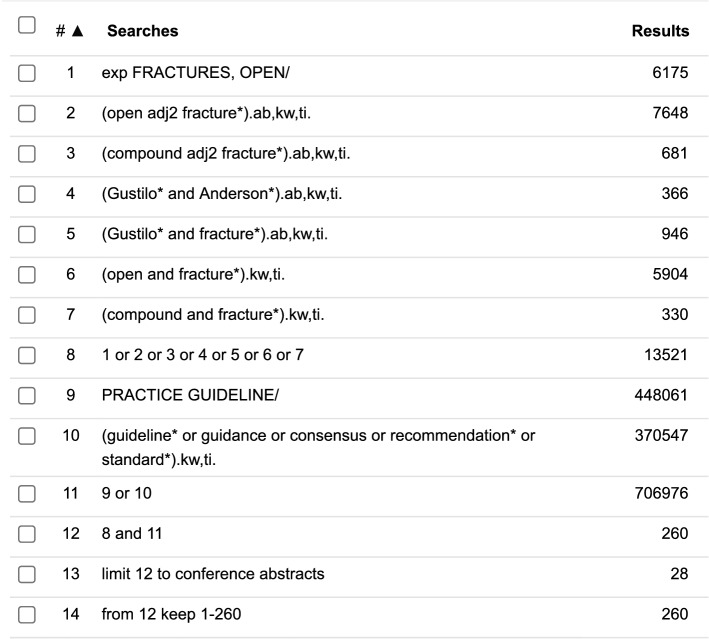
Systematic search strategy

## Appendix

See Fig. 3.
